# Towards an ideotype for food-fuel dual-purpose wheat in Argentina with focus on biogas production

**DOI:** 10.1186/s13068-021-01941-x

**Published:** 2021-04-05

**Authors:** Nadia Gabbanelli, Elisa Erbetta, Maria Eugenia Sanz Smachetti, Máximo Lorenzo, Paola Mónica Talia, Ignacio Ramírez, Magdalena Vera, Ignacio Durruty, Ana Clara Pontaroli, Maria Mercedes Echarte

**Affiliations:** 1 Unidad Integrada Balcarce (Estación Experimental Agropecuaria Balcarce, Instituto Nacional de Tecnología Agropecuaria - Facultad de Ciencias Agrarias, Universidad Nacional de Mar del Plata), CC 276, 7620 Balcarce, Argentina; 2 Instituto de Innovación para la Producción Agropecuaria y el Desarrollo Sostenible (Estación Experimental Agropecuaria Balcarce, Instituto Nacional de Tecnología Agropecuaria – Consejo Nacional de Investigaciones Científicas y Técnicas), CC 276, 7620 Balcarce, Argentina; 3 Instituto de Agrobiotecnología y Biología Molecular (IABIMO), Instituto Nacional de Tecnología Agropecuaria (INTA), Consejo Nacional de Investigaciones Científicas Y Tecnológicas (CONICET), Hurlingham, Buenos Aires, Argentina; 4 Grupo de Ingeniería Bioquímica, INCITAA, Facultad de Ingeniería, Universidad Nacional de Mar del Plata, Mar del Plata, Argentina

**Keywords:** 2G biofuels, Wheat straw, Anaerobic digestion, Straw quality

## Abstract

**Background:**

Wheat straw, one of the most abundant agricultural residues worldwide, can be used to produce biogas, which is considered one of the most efficiently produced renewable energies. Wheat grown with the dual-purpose of producing food and biogas should display simultaneously high grain and straw yields, low lodging susceptibility and high conversion efficiency of straw into biogas. The aims of this study were to determine the best food-fuel dual-purpose wheat candidates among 36 wheat genotypes—including French, CIMMYT and local (*Criollo*) germplasm—used in breeding programs in Argentina and to gain some insights into the relationships between key traits relevant for dual-purpose wheat genotypes.

**Results:**

High variability in individual key traits for dual-purpose wheat ideotype has been found. Genotypes of French origin displayed the highest grain yield, and those of CIMMYT origin, the lowest straw yield. Genotypes of *Criollo* origin showed the highest lodging susceptibility, and French ones, the lowest. Straw yield was positively correlated with grain yield in all genotypes, and negatively correlated with plant height in genotypes of *Criollo* origin. Straw conversion into biogas was measured in terms of the biogas potential production and kinetic parameters *B*_max_ (maximum specific biogas production) and *k* (first-order kinetic constant) were analyzed. All key traits were analyzed together by a principal component analysis. Baguette 31 and SNR Nogal, two genotypes of French origin, showed high grain yield, high-to-very high straw yield, low lodging susceptibility, and moderate-to-high *B*_max_ and *k*. Buck Guapo and Buck Baqueano, two genotypes of *Criollo* origin, displayed good values for grain yield, straw yield and *B*_max_ and *k*. However, their high lodging susceptibility precludes their production in shallow soils or high-input systems. Lastly, some old genotypes (e.g. Klein Atlas) harbored a good combination of all key traits and could prove valuable to be included in future breeding programs for dual-purpose wheat.

**Conclusions:**

While none of the genotypes excelled in every key trait, a few candidates showed potential for dual-purpose ideotype, particularly Baguette 31 and SNR Nogal. The challenge lays in gathering all attributes for food and 2G fuel in the same genotype.

**Supplementary Information:**

The online version contains supplementary material available at 10.1186/s13068-021-01941-x.

## Background

The contribution of fossil fuels to global climate change and the growing demand for energy are the main drivers for worldwide biofuels development [1]. In this context, biomass emerges as one of the most important resources of energy for the sustainable development of societies, in a context of circular economy and environmental protection [2]. Lignocellulosic biomass, the most abundant organic carbon source in the world, is one of the most attractive alternatives for second-generation (2G) biofuel production due to its high and ubiquitous availability and low cost [3, 4]. Straw is a lignocellulosic material largely produced by agriculture, being wheat (*Triticum aestivum* L.) straw one of the most abundant agricultural residues on earth [5]. In Argentina, wheat production is projected to reach a record of 20.2 million tons by 2021 [6], anticipating an approximate amount of available straw of 26.3 million tons [7].

Wheat straw has multiple applications, including use as animal bedding, mushroom-production substrate, and feedstock for biomass-burning power stations [8]. Its supply greatly exceeds its demand, and a large amount of straw is incorporated into the soil after grain harvest [9]. Hence, a significant amount of wheat straw could be used as feedstock for bioenergy production without threatening other straw uses or soil fertility [10]. Different types of energy can be derived from wheat straw through thermochemical or biochemical processes: liquid fuels such as bioethanol, gaseous fuels such as biogas (composed mainly of methane and carbon dioxide), and electricity from direct combustion [11]. Among them, biogas production technology is considered one of the most efficient for producing renewable energy, since it can achieve high energy recovery, and economic and environmental benefits [2, 12]. Recently, an innovative concept of cereals as dual-purpose (DP) for food (wheat is the staple for 35% of the world’s population) and 2G-biofuel was introduced [13]. As mentioned by Townsend et al*.* [14], an ideal genotype (ideotype) for these purposes would be characterized by high grain and straw yields, good lodging resistance and high straw digestibility.

Effects of the environment, the genotype, and their interaction on wheat grain yield (GY) and its components have been largely studied [15–18]. Historically, wheat breeding has been focused on increasing GY per unit area and progress in breeding for GY potential is well documented in the literature [19–22]. Breeding for novel wheat genotypes with reduced plant height has allowed to increase genetic gains and significantly contributed to increase wheat productivity globally [23–26]. Some authors observed that this increase was achieved without a significant change in total aboveground biomass [27–29], while others claimed that GY increases came at the expense of straw yield (SY) [19]. Moreover, some agronomic practices designed to increase GY have resulted in weaker stems and surface roots, and, hence, greater lodging risk [30–32], which in turn impair further GY increase. However, it has been suggested that additional improvements in GY could be achieved by increasing photosynthetic capacity through the optimization of biomass production while maintaining lodging resistance [23].

To successfully manage wheat straw as a commodity and to help position it in the biofuel industry, it is crucial to understand the correlations between biomass, GY, and SY [33], since selecting cultivars with higher SY and without negative effects on GY may constitute a mean to increase the overall straw resource. Even though SY is known to be influenced by several environmental and management factors, including cultivar selection, it is rarely reported in the literature [14, 33–35]. One of the most frequently used approaches to assess SY is based on empirical models that infer SY from GY statistics [36]. However, the uncertainties related to varietal differences in biomass partitioning and its complex response to different environmental and management factors [37] are difficult to address and, therefore, it becomes necessary to record SY in dedicated field trials [36, 38].

Straw digestibility is another relevant trait for DP wheat and it refers to the proportion of fermentable sugars released from lignocellulosic biomass cell wall carbohydrate polymers during hydrolysis. It has been shown to be the rate-limiting step in biomass transformation into bioenergy [39, 40]. Anaerobic digestibility, usually measured in terms of its biogas or biomethane potential defines the quality of the biomass as a feedstock for biogas production [41]. Biogas potential production of wheat straw has been modeled by first-order kinetics [42, 43], where the maximum specific biogas production (*B*_max_) and the first-order kinetic constant (*k*) are used to characterize the suitability of a feedstock for biogas production. According to this model, *B*_max_ represents the maximal biogas that can be produced by volatile solids present in the biomass while *k* estimates the biogas production rate. Kinetic parameters of biogas potential not only define the value of the feedstock for biogas production, but they also give important information for the design and operation of anaerobic digesters [44]. Straw digestibility is often restricted by the complex composition of lignocellulosic material, consisting of three main types of polymers: cellulose, hemicellulose, and lignin. Recalcitrance is determined not only by the wall polymer features but also by the interactions among its components [45–48]. However, no correlation between straw digestibility and GY has been found so far and, hence, it might be possible to improve straw digestibility through breeding without any critical negative effects on GY [49].

The aims of this study were to determine the best food-fuel wheat ideotype candidates among 36 wheat genotypes currently used in breeding programs in Argentina and to give some insights into the relationships between key traits relevant for DP wheat genotypes. To this end, we quantified straw and grain yields and characterized biomass both compositionally and morphometrically. Kinetic parameters of biogas potential production and methane potential yield from straw were determined for all genotypes. Furthermore, correlations among traits were analyzed and genotypes candidates for DP use selected in an effort to assist future breeding programs.

## Results

Thirty-six wheat genotypes carrying germplasm from three different origins (i.e. CIMMYT, *Criollo* and French) were selected and cultivated during seasons 2014 and 2017 (Table 1). Although most genotypes were modern, old genotypes not carrying dwarfing genes (i.e. genotypes 19, 20, 22, 23, 24, 32 and 36) were also included. Under the framework proposed by Townsend et al. [14], key traits of the wheat DP ideotype were analyzed: (i) grain yield and straw yield (“Biomass production and allocation” section); (ii) morphology and lodging susceptibility (“Wheat straw morphology and lodging susceptibility” section), and (iii) biomass composition and biogas potential production (“Straw biomass composition and biogas potential production” section). Finally, key traits were integrated in a principal component analysis (PCA, “Target traits for ideotype design” section) and the best candidates for DP wheat among these genotypes were identified.

**Table 1:** Agronomic characteristics of the wheat genotypes

Origin	Yield group	ID	Genotype	Grain yield (kg/ha)	Straw yield (kg/ha)	Harvest index
CIMMYT	High Yield	1	Buck AGP Fast	5778 ± 37	7762 ± 251	0.43 ± 0.01
		2	Don Mario Arex	5657 ± 368	8233 ± 1378	0.41 ± 0.03
		3	Don Mario Atlax	6050 ± 736	8851 ± 600	0.40 ± 0.01
		4	INIA Centinela	5521 ± 256	9062 ± 1309	0.38 ± 0.02
		5	Klein Don Enrique	5784 ± 300	8675 ± 1298	0.40 ± 0.05
		6	Sursem LE 2331	5767 ± 257	9027 ± 996	0.39 ± 0.02
	Low Yield	7	ACA 907	2337 ± 26	5743 ± 720	0.29 ± 0.03
		8	BIOINTA 1003	2843 ± 67	7901 ± 441	0.27 ± 0.02
		9	BIOINTA 3004	2833 ± 230	4875 ± 109	0.37 ± 0.02
		10	Buck Puelche	2888 ± 112	6361 ± 100	0.31 ± 0.01
		11	Klein Cacique	2945 ± 192	6423 ± 317	0.32 ± 0.03
		12	Klein Yarara	2149 ± 218	4946 ± 187	0.30 ± 0.01
*Criollos*	High Yield	13	BIOINTA 1000	5689 ± 14	9317 ± 223	0.38 ± 0.01
		14	Buck 75 Aniversario	6269 ± 358	9555 ± 288	0.40 ± 0.01
		15	Buck Baqueano	5968 ± 556	11,005 ± 1013	0.35 ± 0.01
		16	Buck Guapo	5989 ± 237	13,156 ± 942	0.31 ± 0.01
		17	Buck Ranquel	5486 ± 403	10,442 ± 129	0.34 ± 0.02
		18	Don Mario Themix^a^	5317	8252	0.38
	Low Yield	19	Barletta 77	2864 ± 466	9437 ± 2064	0.24 ± 0.01
		20	Buck Naposta	2145 ± 348	7686 ± 2985	0.23 ± 0.04
		21	INIA Condor	3503 ± 143	7078 ± 126	0.33 ± 0.01
		22	Klein Impacto	2174 ± 407	6869 ± 1606	0.24 ± 0.01
		23	Klein Rendidor	3059 ± 127	6834 ± 2	0.31 ± 0.01
		24	Oleata Artillero	1628 ± 295	6680 ± 897	0.20 ± 0.01
French	High Yield	25	BSY 100	4922 ± 249	9500 ± 1442	0.34 ± 0.02
		26	Baguette 9	5239 ± 315	8483 ± 1692	0.39 ± 0.03
		27	Baguette 18	5083 ± 267	8622 ± 735	0.37 ± 0.03
		28	Baguette 19	5188 ± 309	8254 ± 391	0.39 ± 0.01
		29	Baguette 31	5691 ± 236	10,308 ± 544	0.36 ± 0.02
		30	SNR Nogal	6367 ± 568	10,787 ± 1003	0.37 ± 0.01
	Low Yield	31	BSY 200^a^	2702	6603	0.29
		32	Klein Atlas	3592 ± 156	8528 ± 63	0.30 ± 0.01
		33	Klein Centauro	4166 ± 120	9879 ± 950	0.30 ± 0.03
		34	Baguette 10	4027 ± 359	6596 ± 505	0.38 ± 0.03
		35	Baguette 21	3946 ± 269	6803 ± 757	0.37 ± 0.01
		36	Sinvalocho	3235 ± 305	7083 ± 1462	0.32 ± 0.02

Origin, yield group, cultivar name, grain yield, straw yield and harvest index of genotypes grown during the 2014 season in Balcarce, Argentina. Data represent the mean and standard error of two replicates

^a^Data represent a single determination

### Biomass production and allocation

Grain yield (GY), straw yield (SY), and harvest index (HI, the ratio between GY and aboveground biomass production) were determined for all genotypes during the 2014 season. Genotypes were split into two groups according to their GY (high yield: over 4000 kg/ha; low yield: less than 4000 kg/ha), and from the combination of the two yield groups and the three origins, six genotype groups were defined: CIMMYT-high yield (CI-HY); CIMMYT-low yield (CI-LY); *Criollo*-high yield (CR-HY); *Criollo*-low yield (CR-LY); French-high yield (FR-HY); French-low yield (FR-LY).

Mean GY, SY and HI obtained across all genotypes during 2014 season were 4300 ± 1485 kg/ha, 8211 ± 1776 kg/ha, and 0.34 ± 0.06, respectively. Significant effects of genotype on all traits were detected (*p* < 0.01) and variability, by means of CV% were 35% for GY, 22% for SY, and 17% for HI. Germplasm origin significantly affected GY (*p* = 0.047), with GY values for genotypes of French origin (4591 ± 218 kg/ha) higher than those of *Criollo* and CIMMYT ones (4117 ± 370 and 4213 ± 333 kg/ha, respectively). Both GY and origin independently affected SY (*p* < 0.01). Mean SY across low-yield genotypes was lower than that of high-yield ones (7029 ± 292 and 9443 ± 274 kg/ha, respectively). Although a positive correlation (*r* = 0.74, *p* < 0.0001) between GY and SY was found, high variability of SY still exists in each genotype group. For instance, when only high-yield genotypes were considered (CV% of GY = 7.3), SY still displayed high variability (CV% = 14.0). Genotypes with CIMMYT origin showed lower SY (7511 ± 356 kg/ha) than C*riollo* and French ones (8894 ± 498 and 8534 ± 359 kg/ha, respectively). By defining the 75th percentile as a threshold value for SY (9317 kg/ha), genotypes 13, 14, 15, 16 and 17 (CR-HY group), and 29 and 30 (FR-HY group) were identified as genotypes with both high GY (high-yield group) and high SY (above the threshold). On a 90th percentile-based threshold (10,442 kg/ha of SY), only 15, 16 and 30 could be regarded as very high SY genotypes.

**Fig. 1:**
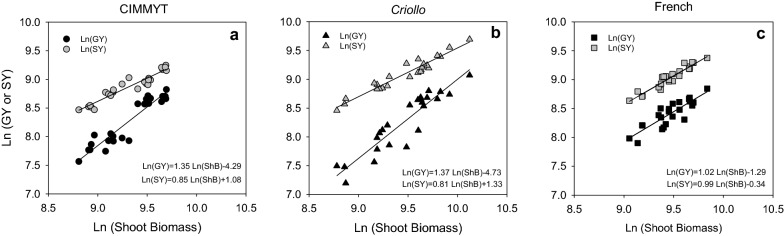
Allometric analyses of wheat biomass. Allometric relations for genotypes with germplasm of different origins: CIMMYT (**a**), *Criollo* (**b**), and French (**c**). Continuous lines represent linear regression between Ln of grain or straw yield and the Ln of shoot biomass. Inset to figures contain linear equations with best fitting parameters for each data set

Origin and GY interacted in their effects on HI (*p* < 0.01): low-yield genotypes had lower HI than high-yield genotypes when considering CIMMYT and *Criollo* origin, while HI did not change with GY for French genotypes. Figure 1 shows biomass allometric analyses for all genotypes according to their origin. Allometric constants (i.e. slopes of relationships in Fig. 1) for CIMMYT and *Criollo* genotypes were significantly higher than 1 for GY and lower than 1 for SY, indicating that allocation to grains increased alongside plant size at the expense of biomass allocation to straw (Fig. 1a, b). For plants of the same size, wheat genotypes from CIMMYT origin displayed higher allocation to grains (and thus, lower to straw) than *Criollo* genotypes, in agreement with HI values, while French genotypes showed an intermediate behavior. Slopes close to 1 indicated that French genotypes conserved their biomass allocation pattern regardless of plant size (Fig. 1c).

### Wheat straw morphology and lodging susceptibility

Plant morphology and lodging susceptibility index (∆LS, Eq. 1) were measured for all genotypes during the 2014 season (see Additional file 1). Genotype effects were significant on all traits measured (*p* < 0.005). Table 2 shows morphometric properties of straw and ∆LS of genotype groups. Interactive effects of GY and germplasm origin were found on stem diameter (*p* = 0.028): low-yield genotypes from CIMMYT origin showed larger stem diameter than high-yield ones, but no differences were observed between yield groups of French or *Criollo* origin. Stem wall thickness was significantly and independently affected by GY and origin (*p* < 0.05): low-yield genotypes showed a thicker stem wall than high-yield ones (0.55 ± 0.01 vs. 0.49 ± 0.01 mm, respectively); CIMMYT genotypes had thicker walls than French ones (0.55 ± 0.02 vs. 0.49 ± 0.01 mm, respectively), and *Criollo* genotypes showed intermediate values (0.53 ± 0.01 mm). Interactive effects of GY and germplasm origin were found on plant height (*p* = 0.009): CIMMYT and *Criollo*low-yield genotypes were taller than high-yield ones, but no differences were observed between yield groups of French origin. Plant height was negatively correlated with SY in genotypes of *Criollo* origin (r = − 0.59, *p* < 0.05), while no correlation between plant height and SY was found in any other genotype. No effects of either origin or GY were observed on spike dry weight (*p* > 0.005).

**Table 2:** Morphometric characteristics and lodging susceptibility of wheat genotype groups

Genotype group	Stem diameter (mm)	Stem wall thickness (mm)	Plan height (cm)	Dry spike weight (g)	Lodging susceptibility index
CI-HY	3.21 ± 0.12^a^	0.51 ± 0.03^ab^	96.2 ± 1.7^a^	2.21 ± 0.13^ab^	23.8 ± 3.0^ab^
CI-LY	3.54 ± 0.05^c^	0.59 ± 0.02^c^	103.8 ± 2.4^b^	2.19 ± 0.17^ab^	23.6 ± 3.5^ab^
CR-HY	3.27 ± 0.05^ab^	0.50 ± 0.02^ab^	97.6 ± 1.7^ab^	2.26 ± 0.11^b^	26.6 ± 3.0^bc^
CR-LY	3.36 ± 0.06^abc^	0.55 ± 0.02^bc^	115.9 ± 2.6^c^	1.91 ± 0.06^a^	32.2 ± 2.4^c^
FR-HY	3.45 ± 0.06^bc^	0.48 ± 0.02^a^	91.4.5 ± 2.7^a^	2.18 ± 0.06^ab^	16.4 ± 2.3^a^
FR-LY	3.39 ± 0.09^abc^	0.51 ± 0.01^ab^	95.2 ± 2.8^a^	2.01 ± 0.11^ab^	16.7 ± 2.4^a^

Stem diameter, stem wall thickness, plant height, dry spike weight, and lodging susceptibility index were determined during the 2014 season. Data represent trait’s mean values ± SE (*n* = 12) of all genotypes grouped according to Table 1. Different letters indicate significant differences (*p* ≤ 0.05)

Germplasm origin was the only factor affecting ∆LS (*p* < 0.01). Genotypes with *Criollo* germplasm were the most susceptible to lodging (∆LS = 29.4 ± 1.9), French genotypes were the most resistant to lodging (∆LS = 16.5 ± 1.6), and CIMMYT ones displayed an intermediate susceptibility (∆LS = 23.7 ± 2.3). Four French genotypes (29, 30, 34 and 35) showed ∆LS below the 10th percentile (∆LS = 8.06) and were considered to have low lodging susceptibility.

For genotypes of CIMMYT origin, higher GY was related to stems with smaller diameter, thinner wall, and lower plant height. *Criollo* genotypes showed differences related to GY only in plant height and spike weight. Lastly, no differences were found between high- and low-yield genotypes of French origin for any of the morphometric traits neither for lodging susceptibility (see Additional file 1).

### Straw biomass composition and biogas potential production

Cell wall main components were determined to characterize straw chemical composition of the 36 genotypes during the 2017 season. Cellulose content ranged between 0.36 and 0.47 mg/mg DW, hemicellulose between 0.27 and 0.33 mg/mg DW, and lignin between 0.12 and 0.21 mg/mg DW (see Additional file 2). Lignin content showed higher variability (CV = 14.4%) than cellulose (CV = 6.5%) and hemicellulose (CV = 5.1%). Table 3 shows mean values of cellulose, hemicellulose, and lignin for the genotype groups. No significant differences were found in cellulose and hemicellulose content among groups. Interactive effects of origin and GY were detected on lignin (*p* = 0.03): higher lignin content was observed in high-yield genotypes from CIMMYT origin, while the opposite effect was observed for French and *Criollo* genotypes.

**Table 3:** Concentration of main components of wheat straw

Genotype group	Cellulose (mg/mg DW)	Hemicellulose (mg/mg DW)	Lignin (mg/mg DW)
CI-HY	0.407 ± 0.003^a^	0.312 ± 0.001^b^	0.175 ± 0.003^b^
CI-LY	0.397 ± 0.006^a^	0.299 ± 0.003^ab^	0.154 ± 0.004^ab^
CR-HY	0.411 ± 0.003^a^	0.303 ± 0.002^ab^	0.140 ± 0.003^a^
CR-LY	0.398 ± 0.005^a^	0.295 ± 0.003^a^	0.163 ± 0.003^ab^
FR-HY	0.404 ± 0.005^a^	0.307 ± 0.003^ab^	0.148 ± 0.001^a^
FR-LY	0.400 ± 0.005^a^	0.302 ± 0.003^ab^	0.162 ± 0.004^ab^

Cellulose, hemicellulose, and lignin were determined in dry straw of the 36 genotypes grown during the 2017 season. Data represent mean ± SE (*n* = 6) of all genotypes grouped according to Table 1. Different letters indicate significant differences (*p* < 0.05)

To assess the performance of the wheat genotypes as bioenergy feedstock, specific biogas production was measured over the 2014 and 2017 seasons. A first-order kinetic equation (Eq. 2) was fitted to experimental data and kinetic parameters *B*_max_ and *k* were determined for each individual genotype as described in “Biogas potential production and kinetic analysis” section (see Additional file 3 for individual genotype information). Mean values for the best fitting kinetic parameters, methane content in biogas, and methane yield (MY) for the genotype groups are presented in Table 4. A significant effect of the season on *B*_max_ (*p* = 0.002) was observed, with average values higher for season 2017 (433.1 ± 2.8) than for 2014 (420.7 ± 2.7). Additionally, there were interactive effects of genotype and season on *B*_max_ and *k* (*p* < 0.0001). Although the best genotypes for each kinetic parameter were different in each season, some genotypes were consistent in both seasons: genotypes 16, 18, 25, 29 and 36 exceeded the 75th percentile for *B*_max_ (436.0 Nml/g VS for season 2014 and 447.0 Nml/g VS for season 2017), while genotypes 6, 30, 34 and 35 had *k* values higher than the 75th percentile (0.132 day^−1^ for season 2014 and 0.140 day^−1^ for season 2017). Noteworthy, genotypes 6 and 18 showed values above the 75th percentile for both kinetic parameters during the 2017 season.

**Table 4:** Kinetic parameters of biogas production, methane (CH_4_) percentage and methane yield

Season	Group	*B*_max_ (Nml/g VS)	*k* (day ^−1^)	CH_4_ (%)	Methane yield (m^3^/ha)
2014	CI-HY	424.5 ± 5.8^b^	0.111 ± 0.004^a^	55.8 ± 0.5^a^	1800 ± 29^c^
	CI-LY	418.2 ± 5.5^ab^	0.125 ± 0.005^b^	55.3 ± 0.5^a^	1238 ± 66^a^
	CR-HY	422.8 ± 6.8^ab^	0.126 ± 0.002^b^	55.1 ± 0.4^a^	2164 ± 137^d^
	CR-LY	405.6 ± 9.5^a^	0.118 ± 0.004^ab^	56.1 ± 0.5^a^	1503 ± 54^b^
	FR-HY	426.7 ± 4.8^b^	0.127 ± 0.003^b^	55.5 ± 0.5^a^	1944 ± 65cd
	FR-LY	426.4 ± 4.8^b^	0.124 ± 0.005^b^	55.6 ± 0.5^a^	1562 ± 73^b^
2017	CI-HY	432.5 ± 6.7^ab^	0.128 ± 0.003^a^	55.6 ± 0.2^a^	nd
	CI-LY	417.0 ± 4.4^a^	0.126 ± 0.003^a^	56.0 ± 0.3^a^	nd
	CR-HY	442,4 ± 6.4^b^	0.129 ± 0.006^a^	55.3 ± 0.2^a^	nd
	CR-LY	439.4 ± 8.3^b^	0.122 ± 0.008^a^	55.9 ± 0.2^a^	nd
	FR-HY	434.3 ± 6.6^ab^	0.126 ± 0.006^a^	55.8 ± 0.2^a^	nd
	FR-LY	432.1 ± 7.1^ab^	0.127 ± 0.005^a^	55.9 ± 0.3^a^	nd

Best fitting values for *B*_max_ and *k*, and methane content of biogas for the six wheat genotype groups, for both 2014 and 2017 seasons. Methane content was determined after cumulative biogas reached a plateau. Methane yield was determined during season 2014. Data represent mean ± SE (*n* = 12) of all genotypes grouped according to Table 1. Different letters indicate significant differences (*p* ≤ 0.05)

*nd* not determined

No effects of GY nor origin were detected on the kinetic parameters in either season. Methane content did not differ among genotypes or seasons and showed a mean value of 51.1% (v/v). MY (Eq. 3), was strongly correlated to SY (*r* = 0.96, *p* < 0.001) and, consequently, was affected by GY (*p* < 0.001) and origin (*p* < 0.001). Genotypes from CIMMYT origin presented lower MY than French and *Criollo* ones, while high-yield genotypes presented higher MY than low-yield ones, regardless of their origin. Noteworthy, despite the high correlation between MY and SY, some high SY genotypes (13, 14 and 19) were not considered high MY genotypes, since their MY values were below the 75th percentile (1880 m^3^/ha). Genotype 16 stood out for its high MY as a consequence of its high SY and *B*_max_ (see Additional file 3 for individual genotype information).

**Fig. 2:**
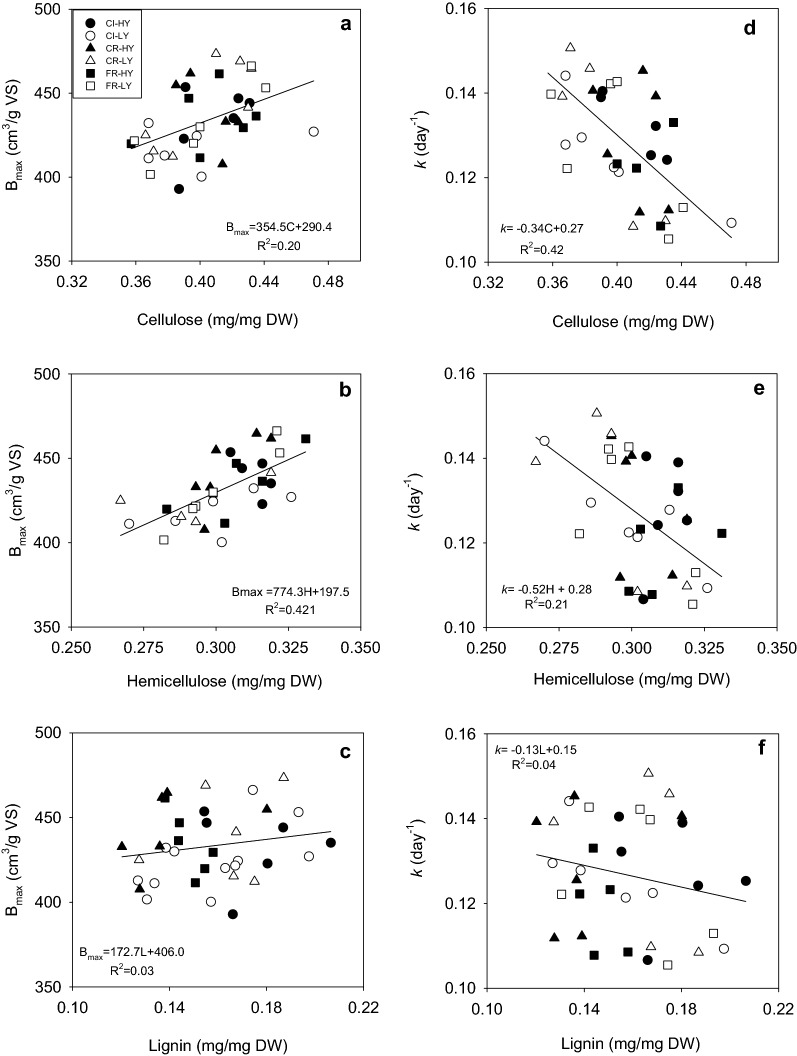
Relationship between straw main components and biogas kinetic parameters for wheat genotypes. Linear regressions between biogas kinetic parameters: *B*_max_ (**a**–**c**) and *k* (**d**–**f**), and biomass components: cellulose (**a**, **d**), hemicellulose (**b**, **e**), and lignin (**c**, **f**) were performed for the 36 wheat genotypes. Continuous lines represent the fitting of the linear regressions and inset to figures contain equations with best fitting parameters for each data set

To evaluate potential relationships between biogas kinetic parameters (*B*_max_ and *k*) and the main components of straw biomass (cellulose, hemicellulose, and lignin), regression analyses were performed (Fig. 2). Genotypes with higher cellulose and hemicellulose content showed a tendency to have higher values of *B*_max_, while lignin content effect on it was not significant (Fig. 2a–c). Cellulose and hemicellulose content were negatively related to *k* (Fig. 2d, e), while no relationship was observed between lignin content and *k*.

**Fig. 3:**
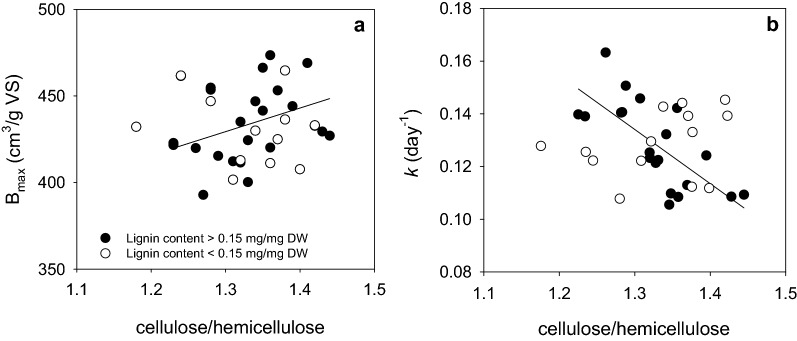
Relationship between kinetic parameters and cellulose-to-hemicellulose ratio. Biogas kinetic parameters *B*_max_ (**a**) and *k* (**b**) as a function of the cellulose-to-hemicellulose ratio. Continuous line represents the fitting of a linear regression to all data (**a**) or to data from genotypes with lignin content higher than 0.15 mg/mg DW (**b**)

To determine the effects of cell wall component interactions on biogas kinetic parameters, correlation analyses were performed between cellulose-to-hemicellulose, cellulose-to-lignin, and hemicellulose-to-lignin ratios and *B*_max_ or *k*. As no significant correlation was found (*p* > 0.05, data not shown), a cluster analysis of these correlations for genotype groups according to their lignin content was performed. Regression analyses between cellulose-to-hemicellulose ratio and both kinetic parameters were performed for each lignin content, either above or below 0.15 mg/mg DW. Although the determination coefficient value was too low to explain all data variation (*R*^2^ = 0.13), a positive relationship between cellulose-to-hemicellulose ratio and *B*_max_ was observed, regardless of the lignin content (*p* = 0.0347). In opposition, a negative relationship between cellulose-to-hemicellulose ratio and *k* (*R*^2^ = 0.51; *p* < 0.0001) was found for genotypes with lignin content higher than 0.15 mg/mg DW (Fig. 3).

### Target traits for ideotype design

**Fig. 4:**
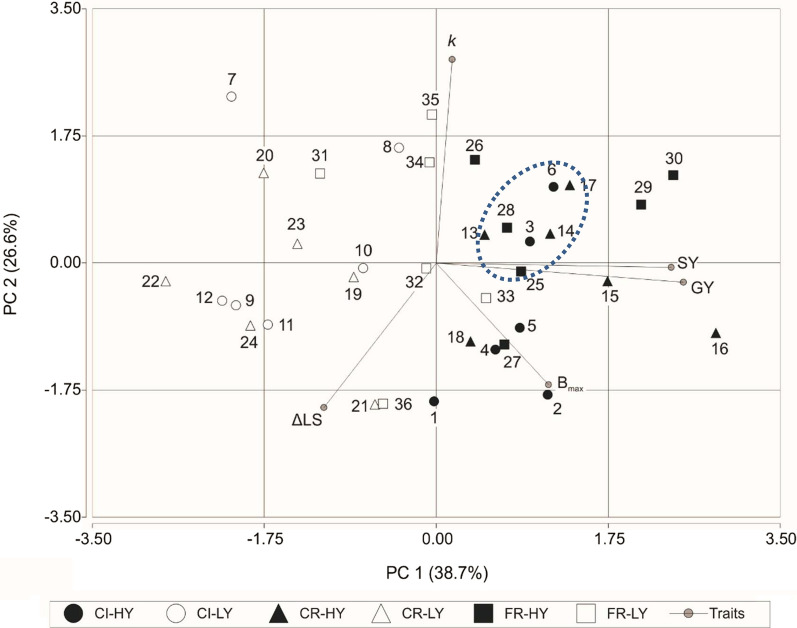
Principal Component Analysis for key traits for the wheat dual-purpose ideotype. A Principal Component Analysis (PCA) was performed based on GY, SY, lodging susceptibility, and biogas production kinetic parameters for the 36 wheat genotypes (see Table 1 for numeric reference). Correlation of traits with PC1 were 0.913, 0.868, 0.415, − 0.415 and 0.06 for GY, SY, *B*_max_, ∆LS, and *k,* respectively. Correlation of traits with PC2 were 0.843, − 0.599, − 0.504, − 0.080 and − 0.018 for *k*, ∆LS, *B*_max_, GY, and SY, respectively. Ellipse was empirically built

The ideotype for food and 2G-fuel DP wheat should bear simultaneously high GY, SY, *B*_max_, and *k* and low ∆LS. In the previous sections, key traits were separately analyzed. In the present section, a principal component analysis (PCA) was performed to analyze all key traits combined (Fig. 4). Grain and straw yields, *B*_max_, and ∆LS accounted for principal component 1 (PC1), explaining 38.7% of the variability of the data. Principal component 2 (PC2) represented 26.6% of the variability and was mainly composed by *k,* *B*_max_, and ∆LS. A large variation in both PCs was observed among the genotypes evaluated. Low-yield genotypes (empty symbols in Fig. 4) presented the lowest values of PC1, while high-yield genotypes (black symbols in Fig. 4) were located to the right side of the bi-plot and had the highest PC1 values. Genotypes of French and *Criollo* origins presented the highest values of PC1. Noteworthy, no significant relation with GY and SY was found for the kinetic parameters of biogas production or lodging susceptibility in this analysis.

A closer analysis of the PCA in combination with the results mentioned in the previous sections showed the presence of genotypes with outstanding performance in some of the traits but not in all. Among the three genotypes with high GY and very high SY (“Biomass production and allocation” section), genotype 15-Buck Baqueano produced biogas with high *k* and genotype 16-Buck Guapo with very high *B*_max_ (above 90th percentile), but both displayed high lodging susceptibility (∆LS above 75th percentile). Genotype 30-SNR Nogal showed very high SY, low ∆LS, high *k* value and moderate *B*_max_ (above the 50th percentile). Among the genotypes with the lowest ∆LS (“Wheat straw morphology and lodging susceptibility” section), genotype 29-Baguette 31 showed high GY, SY, and *B*_max_ and moderate *k* (above the 50th percentile), genotype 34-Baguette 10 had high *k* but low GY and SY, and genotype 35-Baguette 21 had high *k* but low GY, SY and low *B*_max_ values. Finally, among the genotypes with high values for the kinetic parameters (“Straw biomass composition and biogas potential production” section), genotypes with high *k* values (7-ACA 907, 26-Baguette 9 and 35-Baguette 21) did not show any other suitable trait, while among those with high *B*_max_, genotype 16-Buck Guapo was a high GY and SY genotype that displayed low lodging resistance and low *k*.

The dotted circle in Fig. 4 defines an area where acceptable values for all traits can be expected. Among them, genotypes 6-Sursem LE 2331 and 28-Baguette 19 were found to have values above the 50th percentile for all traits (or below for ∆LS). Close to this cluster, two genotypes not carrying dwarfing genes (32-Klein Atlas and 33-Klein Centauro) showed relatively good traits for DP wheat.

## Discussion

In this work, we have addressed genotype effects on relevant traits for the production of wheat with the dual-purpose of food (grains) and 2G-biofuel (straw); more specifically, for biogas production. We studied 36 genotypes with contrasting GY that included old and modern cultivars bearing germplasm of 3 different origins (CIMMYT, *Criollo* and French). In agreement with previous reports, we found high variability in all agronomic traits (GY, SY, HI, and ∆LS) [35, 38, 50, 51]. When all genotypes were regarded together, we found a positive correlation between GY and SY, in agreement with other authors [52], which suggests that it is possible to increase SY without compromising GY, a central issue for developing DP cultivars [14]. However, despite this positive correlation, high variability of SY can still be found in groups of similar GY (Table 1, [35]). These discrepancies regarding SY and GY correlation point out the importance of gaining knowledge on biomass production and allocation in wheat plants [38].

Harvest index (HI) indirectly indicates the partitioning of biomass between grain and straw [53] and a number of studies have demonstrated that HI is affected by genetic variations [52, 54]. Both SY and biomass allocation pattern differed among groups of genotypes of the same origin (Table 1 and Fig. 1). To our knowledge, this is the first time that origin effects on wheat biomass allocation are reported. In genotypes of French origin, both grain and straw yields increased at the same rate with plant size, but for genotypes of CIMMYT and *Criollo* origins, higher GY was attained at the expense of SY, consistently with previous reports [19, 55]. According to this, breeding for bigger plants from CIMMYT or *Criollo* origin do not grant higher SY, while it could be effective to increase both GY and SY in French origin genotypes.

Plant height was significantly higher for low-yield genotypes of CIMMYT and *Criollo* origins, while no effect of GY on plant height was detected on genotypes of French origin. A common strategy to increase GY potential has been to increase the HI through plant height reduction [21, 56]. However, in the last 30 years, a raise in GY potential without changes in plant height has been achieved in countries such as the United Kingdom [57] and Argentina [51], and even a slight increase in plant height with GY progress in modern CIMMYT cultivars has been reported [58]. Plant height has been shown to be positively correlated [52, 59] and not correlated at all [34] with SY. Here, we found that the association between plant height and SY differed among genotypes of different germplasm origin: while a negative correlation exists in genotypes of *Criollo* origin, no correlation was found in genotypes of the other two origins.

Morphometric traits measured at maturity were combined in a simple model to evaluate cultivar lodging susceptibility in low lodging conditions [60]. Lodging tolerance has been directly associated to plant height [61] and the risk of lodging was notably reduced after the introgression of dwarfing genes [62–64]. However, we found a moderate correlation between plant height and lodging susceptibility (*r* = 0.60, *p* < 0.05). Genotypes carrying French germplasm showed the lowest lodging susceptibility while *Criollo* ones showed the highest (Table 2). When analyzed by genotype groups, we found that correlations between height and lodging were diverse, ranging from positive (e.g. for FR-HY genotypes) to negative (e.g. for CI-HY). This observation highlights the variability of plant structure and its response to genetic factors. It also points out to the importance of complementing morphology observations with biomass composition in order to understand complex traits such as lodging. For example, for CI-HY genotypes, where there is a negative correlation between plant height and lodging susceptibility, lignin content might play a key role in avoiding lodging in plants with heavier spikes. In this sense, breeding targeted to increase capsizing resistance has led to a decrease in the saccharification potential of straw due to changes in the stem components [65]. In contrast, lodging avoidance among CR-HY genotypes with increasing plant height could be explained by thicker stems with thicker walls rather than by changes in lignin content. Finally, for FR-HY genotypes lodging avoidance strategy might be in between CIMMYT and *Criollo* cultivars, presenting wider stems and thicker walls in combination with a moderate increase in lignin content.

Many efforts have been done to characterize the factors affecting total biomass of modern wheat cultivars [3, 16, 66, 67]. However, less attention has been paid to factors affecting wheat straw [52]. With the development of 2G-biofuel production, efforts on increasing the yield and quality of straw to maximize the overall value of wheat crop are expected to increase [35, 68]. An analysis run on GY and HI from many trials in the United States allowed to detect differences in SY among wheat classes [33] and, here, we report genotype effects on both SY and composition, in agreement with previous reports [35, 38, 69]. However, to assess the overall potential of biogas production, both SY and its digestibility must be considered. Even though SY has been shown to be the main driver of methane yield (MY) and thus, it has been considered the main factor in feedstock selection for bioenergy production [61], straw digestibility drives the efficiency of the transformation process and determines the inputs it requires, conditioning its overall economic revenue [50, 70–73]. Straw conversion into biogas was assessed using two kinetic parameters, *B*_max_ and *k*. *B*_max_ ranged between 368–459 cm^3^/g VS and 392–473 cm^3^/g VS for 2014 and 2017 seasons, respectively (Additional File 3: Table S3), in agreement with previously reported values [74]. Although MY was strongly correlated with SY, there were some genotypes that showed high SY but low MY, due to low *B*_max_ values (e.g. 13-BIOINTA 1000, 14–Buck 75 Aniversario and 19-Barletta 77). Conversely, some genotypes attained high MY despite not having high SY, due to their high *B*_max_ values (e.g. 2-Don Mario Arex and 27-Baguette 18). In the same sense, genotype 16-Buck Guapo, combining high SY and high *B*_max_, stood out as the best in terms of MY. For biogas production, increasing *B*_max_ reduces biomass transport, handling, storage, and pretreatment costs, while increasing *k* reduces feedstock pretreatments needs, and lowers retention time during anaerobic digestion, allowing reactor downsizing and the possibility of performing the anaerobic digestion at lower temperatures [40].

Biomass digestibility is often restricted by the recalcitrant nature of lignocellulose [75] and is related to lignin content and composition, cellulose crystallinity, hemicellulose content and interaction between cell wall components [45, 76–78] Here, straw composition ranged between 0.36 and 0.46 mg/mg DW for cellulose, 0.27 and 0.33 mg/mg DW for hemicellulose, and 0.12 and 0.21 mg/mg DW for lignin (Additional File 2: Table S2), in agreement with previous reports [75, 79, 80]. We found that biogas production potential and rate were affected by the amount of cellulose and hemicellulose in the biomass, but not by lignin content (Fig. 2). Although lignin content has been associated to lower biogas yield [81–83], a low correlation of biogas potential and lignin has been reported [84]. The limited digestibility of lignin under anaerobic conditions could explain these results [85]. Additionally, the efficiency of cellulose and hemicellulose degradation into simple sugars has been reported to be dependent on the polysaccharide-to-lignin ratio, among other factors [46]. We found a negative relationship between cellulose-to-hemicellulose ratio and *k* for genotypes with lignin content higher than 15% (w/w, Fig. 3), suggesting a lignin threshold value at which relative content of structural carbohydrates have an effect on biogas production rate. Li et al*.* [77] showed that lignin caused a more severe inhibition on methane production from cellulose than from hemicellulose mainly because lignin-incrusted cellulose cannot be reached by cellulases [86]. Additionally, hemicellulose has been shown to reduce lignocellulose crystallinity and, therefore, to increase biomass digestibility [45]. Hence, predominance of hemicellulose in the biomass could counteract lignin’s inhibitory effect on cellulose degradability on wheat straw with lignin contents higher than the threshold value. At lignin contents lower than this threshold, differences in *B*_max_ and *k* among genotypes could respond to cellulose’s inherent recalcitrance given by its degree of polymerization and crystallinity, and not by the interaction between cellulose and lignin [48]. Accordingly, Dandikas et al*.* [83] found that *k* was not correlated with lignin in biomass with less than 4% of lignin.

We found significant genetic variability in the four traits relevant for a DP wheat ideotype. Although this should facilitate the selection for improved individual traits, trade-offs between some of them require a more complex analysis when looking for the best cultivars currently available. For instance, increasing SY and digestibility could potentially have negative impacts on GY and lodging resistance, hence, reducing the feasibility of a single ideotype [68]. Using percentile-based thresholds, we identified genotypes bearing high GY and SY (e.g. genotype 15-Buck Baqueano, 16-Buck Guapo, 29-Baguette 31 and 30-SNR Nogal). However, among them, only genotypes 29-Baguette 31 and 30-SNR Nogal displayed high lodging resistance. The use of graphical analyses such as the PCA allowed highlighting the strengths and weaknesses of each genotype. For example, when only considering MY, genotype 16-Buck Guapo would stand out as the best candidate. However, low lodging resistance (∆LS = 29.5) would preclude its production in shallow soils or high-input level conditions. It would be interesting to explore whether it is possible to increase lodging resistance of genotype 16-Buck Guapo background without significantly affecting the remaining traits. When considering all traits together, genotypes 29-Baguette 31 and 30-SNR Nogal, both of French origin, emerged as the most promising among all genotypes explored. Interestingly, genotype 6-Sursem LE 2331 showed acceptable values for all traits and might represent an interesting genotype to be used as a starting point to improve CIMMYT origin genotypes for DP use.

By including low-yield genotypes in this study, it was possible to detect genotypes that, despite presenting low GY, SY and *B*_max_, had good values for attributes like *k* or ∆LS (e.g. genotype 7-ACA 907). Also included in this study were old genotypes not carrying dwarfing genes derived from *Criollo* or French origin. They showed both low GY and SY values with variable ∆LS, *k* and *B*_max_. Yet, genotypes like 32-Klein Atlas and 33-Klein Centauro could be of interest in future breeding programs, since they harbor a good combination of all key traits for DP wheat. Old wheat cultivars have been proposed to be particularly promising for climate change mitigation in organic and low input systems, since they could represent a valuable resources more suitable to adapt to future scenarios of sustainable agriculture in a climate change context [87, 88].

Engineering of wheat to produce the ideal DP appears as an attractive strategy to improve the overall economic revenue of the 2G-biofuel production process. In this context, we have analyzed already available wheat cultivars for their potential use as a feedstock for anaerobic digestion without compromising GY. Results here reported can contribute to guide breeding programs meant to improve wheat as a DP crop.

## Conclusion

There is high variability in the key traits for DP wheat ideotype for food and 2G-biofuel. None of the genotypes excels in every key trait, but a few candidates presented acceptable values in many of them. Particularly, two genotypes of French origin (29-Baguette 31 and 30-SNR Nogal) showed high GY, high-to-very high SY, low ∆LS, and moderate-to-high *B*_max_ and *k*. The challenge lays in attaining a genotype with excelling values in all key traits. Breeding efforts in this direction will increase the resources available for lignocellulosic biofuel production without compromising existing food production.

## Methods

### Plant material

Thirty-six genotypes of wheat (*Triticum aestivum* L.) were grown during the 2014 and 2017 seasons. The selected genotypes are a sample of genetic materials used in the past and/or present by breeding programs in Argentina and included French, CIMMYT and local (*Criollo*) germplasm (French, CIMMYT and *Criollo* origin, respectively). Genotypes were chosen based on available information on GY and HI in order to span a wide range of variability for these traits [51, 89]. Old cultivars that do not have dwarfing genes (i.e. 19-Barletta 77; 20-Buck Naposta; 22-Klein Impacto; 23-Klein Rendidor; 24-Oleata Artillero; 32-Klein Atlas and 36-Sinvalocho) were also included to explore biomass attributes that could have been lost in more modern high-yield cultivars.

### Site and crop details

Field experiments were carried out at Balcarce Agricultural Experimental Station of Instituto Nacional de Tecnología Agropecuaria (INTA), Argentina, (37° 84′ 50″ S; 55° 81′ 80″ W; 130 m.a.s.l.). Soil was a complex of fine mixed Typic Argiudoll and fine thermic Petrocalcic Paleudoll (petrocalcic horizon at 140 cm). Experiments were conducted under conventional tillage, with genotypes arranged in randomized complete blocks with two replications. Plots comprised seven rows of 5.5 m in length with a distance between rows of 0.2 m. Genotypes were sown on recommended dates and at recommended densities to achieve optimal yield according to plant lifecycle duration. Soil was fertilized with 25 kg/ha of phosphorous before sowing, and with 300 kg/ha of nitrogen, distributed in two moments, at tillering and at the beginning of the stem elongation period. Experiments were conducted under no nutrient or water limitations. Weeds, pests, and fungal diseases were chemically controlled.

### Agronomic characterization

Plants were harvested at physiological maturity and dried at 60 °C for 48 h. To determine grain and straw yields, the five central rows of the plot cultivated during the 2014 season were mechanically harvested. After shoot biomass determination, spikes and grains were separated from straw and weighed. Straw dry weight was calculated as the difference between shoot biomass and grain weight. All weights were expressed on an area basis. HI was calculated as the quotient between grain weight and shoot biomass weight.

### Lodging susceptibility

Lodging susceptibility was estimated as in Mirabella et al*.* [60]. Plant height was determined as the distance from the ground to the tip of the spike and calculated as the average of two measurements per plot. Stem diameter and stem wall thickness were measured using a digital caliper at 1 cm below the first internode. Lodging susceptibility was calculated according to the following equation:

1 $$\Delta {\text{LS}} = 0.87\Delta H + 27.5\Delta {\text{SW}} - 3.62\Delta J_{x} ,$$

where ∆LS represents deviation of the lodging susceptibility of the ith genotype from the mean value across all genotypes (∆LS = LS_*i*_ – LS), ∆H, ∆SW and ∆*J*_*x*_ are the deviations of plant height (*H*), spike weight and the inertia moment of the area of the stem base values of individual genotypes from the mean traits, respectively. To transform ∆LS to positive values, values were corrected by adding the minimum ∆LS value obtained.

### Biomass allocation

Allometric relationships were calculated as the linear regression between the natural logarithms of GY or SY and the natural logarithm of total plant biomass [90]. The slope of this relationship is called allometric constant (m) and it is the ratio of the logarithmic growth rates of the two components under study. Any value of m other than unity implies a discrepancy between these two rates [91].

### Biomass analysis

Wheat straw samples were obtained by cutting ten consecutive plants above soil surface and removing the spikelets. Plants were dried at 65 °C until constant weight and then milled and sieved through a 2-mm mesh. Total solids, ash content, volatile solids (VS), and lignin content were determined according to NREL Biomass Analytical Methods [92–95]. Acid detergent fiber (ADF), neutral detergent fiber (NDF), and acid detergent lignin (ADL) were determined using the Ankom filter bag system (Ankom Technology, Fairport, NY, USA) [96]. Cellulose was calculated as the difference between ADF and ADL and hemicellulose as the difference between NDF and ADF.

### Biogas potential production and kinetic analysis

Biogas potential production was assessed by performing batch anaerobic digestion experiments in 120 cm^3^ capacity bottles. The inoculum used consisted of equal parts (in VS) of chicken manure, pig slurry and active sludge from a potato-industry biodigester, stabilized as described by Steinmetz et al*.* [97]. Samples and inoculum were mixed at 1:2 ratio on a VS basis. Basal medium containing all the necessary micro- and macro-nutrients for an optimum anaerobic microbial growth was added to all bottles [98]. To maintain anaerobic conditions, the bottles were flushed with CO_2_ and then sealed with rubber stoppers and screwcaps. Anaerobic digestion was run at 37 °C in a water bath with daily manual stirring. Each sample was digested in duplicate. As a negative control, biogas production by the inoculum without any substrate added was measured and later subtracted from the biogas values obtained in each bottle. As a positive control, microcrystalline cellulose (Biopack) was used as a standard substrate.

The biogas volume was estimated by measuring each bottle´s inner pressure with an electronic barometer (Digital high-pressure manometer, Testo 512), and then normalized to 0 °C and 101.3 kPa. Methane content was determined by transferring 5 cm^3^ of the bottle’s headspace gas (v1) into an Einhorn’s saccharimeter containing 1 N NaOH with a syringe. After CO_2_ and H_2_S scrubbing in NaOH, the gas in the saccharimeter (v2) was considered to be methane. The ratio v2/v1 provided the proportion of methane (CH_4_%) in the biogas occupying the bottle’s headspace [98, 99]. Determinations were done after cumulative biogas reached a plateau.

A first-order kinetics model was chosen to describe biogas production over time [100], fitting the data to the exponential equation:

2 $$B = B_{{{\text{max}}}} \times \left( {1 - e^{ - kt} } \right),$$

where *B* represents cumulative biogas, *B*_max_ is the maximum specific biogas production, *k* is the first-order kinetic constant (which allows to estimate biogas production rate) and t the digestion time.

Methane yield (MY) was calculated according to the following equation:

3 $${\text{MY}} = {\text{SY}} \times {\text{VS}} \times B_{\max } \times {\text{CH}}_{4} \% ,$$

where SY represents straw yield, VS are the volatile solids, *B*_max_ is the maximum specific biogas production, and CH_4_% is the percentage of methane present in the biogas.

### Data analysis

Genotypes were split into two groups according to their GY (HY: higher than 4000 kg/ha; LY: lower than 4000 kg/ha^−^). From the combination of two yield groups and three origins (CIMMYT, *Criollo* and French), six genotype groups were defined as indicated in Table 1. Data were analyzed using a multifactorial ANOVA analysis [101]. Factors were germoplasm origin (CIMMYT, *Criollo* and French) and yield group (high and low yield). Differences between treatments were evaluated by the Fisher´s test (*p* < 0.05). Linear and first-order kinetic equations were fitted by non-linear simple regressions using the software Sigmaplot 10.0 [102]. Principal Component Analysis was performed using InfoStat [101].

## Supplementary Information

**Additional file 1.** Morphometric characteristics and lodging susceptibility of the wheat genotypes.

**Additional file 2.** Concentration of main components of wheat straw.

**Additional file 3.** Kinetic parameters of biogas production and methane (CH_4_) percentage for wheat genotypes.

## Data Availability

All data supporting the conclusions of this article are included within the article and in Additional files.

## Abbreviations

2G: Second generation
ADF: Acid detergent fiber
ADL: Acid detergent lignin
*B*_max_: Maximum specific biogas production
CI-HY: CIMMYT-high yield
CI-LY: CIMMYT-low yield
CR-HY: *Criollo-*High yield
CR-LY: *Criollo*-Low yield
CV: Coefficient of variation
DP: Dual-purpose
FR-HY: French-high yield
FR-LY: French-low yield
GY: Grain yield
H: Plant height
HI: Harvest index
MY: Methane yield
*k*: First-order kinetic constant
NDF: Neutral detergent fiber
PC1: Principal component one
PC2: Principal component two
PCA: Principal component analysis
SY: Straw yield
VS: Volatile solids
∆LS: Lodging susceptibility
