# Supplementary material for: Towards an ideotype for food-fuel dual-purpose wheat in Argentina with focus on biogas production
Source: Biotechnol Biofuels. 2021 Apr 5;14:85. doi: 10.1186/s13068-021-01941-x (PMC8022367; doi:10.1186/s13068-021-01941-x)
Supplement: Supplementary file 1 — Additional file 1. Morphometric characteristics and lodging susceptibility of the wheat genotypes. [file 13068_2021_1941_MOESM1_ESM.docx]

**Additional File 1: Morphometric characteristics and lodging susceptibility of the wheat genotypes**

| Origin | Yield group | Genotype | Stem Diameter  (mm) | Stem wall thickness  (mm) | Plant height  (cm) | Dry spike weight  (g) | Lodging susceptibility  index |
| --- | --- | --- | --- | --- | --- | --- | --- |
| CIMMYT | High yield | Buck AGP Fast^a^ | 2.87^a^ | 0.430^a^ | 95.7 ± 0.7 | 2.509 ± 0.360 | 37.3 ± 8.8 |
|  |  | Don Mario Arex | 3.59 ± 0.12 | 0.561 ± 0.030 | 103.2 ± 5.3 | 2.045 ± 0.103 | 19.6 ± 6.9 |
|  |  | Don Mario Atlax^a^ | 3.75^a^ | 0.675^a^ | 101.2 ± 1.7 | 2.124 ± 0.043 | 13.5 ± 0.6 |
|  |  | INIA Centinela | 3.49 ± 0.10 | 0.559 ± 0.006 | 90.0 ± 0.5 | 2.846 ± 0.111 | 28.4 ± 2.1 |
|  |  | Klein Don Enrique | 2.74 ± 0.04 | 0.400 ± 0.007 | 94.0 ± 3.0 | 2.111 ± 0.203 | 28.5 ± 7.2 |
|  |  | Sursem LE 2331 | 2.81 ± 0.01 | 0.432 ± 0.015 | 92.7 ± 3.2 | 1.627 ± 0.212 | 15.5 ± 1.9 |
|  | Low yield | ACA 907 | 3.68 ± 0.13 | 0.556 ± 0.017 | 95.7 ± 1.7 | 2.633 ± 0.370 | 24.3 ± 9.8 |
|  |  | BIOINTA 1003 | 3.60 ± 0.17 | 0.595 ± 0.056 | 103.7 ± 1.2 | 1.825 ± 0.370 | 9.4 ± 9.4 |
|  |  | BIOINTA 3004 | 3.70 ± 0.08 | 0.722 ± 0.045 | 100.5 ± 0.0 | 2.192 ± 0.395 | 35.9 ± 8.9 |
|  |  | Buck Puelche | 3.35 ± 0.05 | 0.619 ± 0.042 | 95.2 ± 0.2 | 2.081 ± 0.098 | 17.5 ± 2.4 |
|  |  | Klein Cacique | 3.47 ± 0.07 | 0.512 ± 0.013 | 112.2 ± 2.2 | 2.200 ± 0.125 | 34.5 ± 4.8 |
|  |  | Klein Yarara | 3.45 ± 0.10 | 0.554 ± 0.025 | 115.2 ± 1.7 | 1.457 ± 0.084 | 19.92 ± 0.4 |
| *Criollos* | High yield | BIOINTA 1000 | 3.11 ± 0.06 | 0.501 ± 0.013 | 102.2 ± 0.7 | 2.257 ± 0.322 | 33.4 ± 7.9 |
|  |  | Buck 75 Aniversario | 3.13 ± 0.10 | 0.484 ± 0.015 | 92.0 ± 1.5 | 2.033 ± 0.240 | 19.3 ± 4.1 |
|  |  | Buck Baqueano | 3.48 ± 0.04 | 0.502 ± 0.020 | 94.2 ± 0.2 | 2.656 ± 0.251 | 29.1 ± 5.5 |
|  |  | Buck Guapo | 3.53 ± 0.12 | 0.606 ± 0.021 | 106.0 ± 1.5 | 2.353 ± 0.480 | 29.5 ± 9.5 |
|  |  | Buck Ranquel | 3.26 ± 0.09 | 0.524 ± 0.020 | 92.5 ± 0.5 | 1.929 ± 0.018 | 14.8 ± 0.9 |
|  |  | Don Mario Themix | 3.08 ± 0.01 | 0.405 ± 0.019 | 98.7 ± 5.8 | 2.319 ± 0175 | 33.6 ± 9.0 |
|  | Low yield | Barletta 77 | 3.38 ± 0.04 | 0.538 ± 0.053 | 112.7 ± 0.2 | 1.818 ± 0.041 | 27.6 ± 1.2 |
|  |  | Buck Naposta | 3.31 ± 0.15 | 0.521 ± 0.003 | 111.2 ± 2.7 | 1.671 ± 0.208 | 24.5 ± 7.1 |
|  |  | INIA Condor | 3.54 ± 0.12 | 0.661 ± 0.024 | 111.2 ± 0.2 | 2.129 ± 0.020 | 27.7 ± 0.7 |
|  |  | Klein Impacto | 3.02 ± 0.06 | 0.499 ± 0.016 | 120.7 ± 2.7 | 1.854 ± 0.086 | 41.7 ± 0.5 |
|  |  | Klein Rendidor | 3.28 ±0.02 | 0.435 ± 0.012 | 107.0 ± 3.0 | 2.055 ± 0.131 | 31.5 ± 5.6 |
|  |  | Oleata Artillero | 3.63 ±0.13 | 0.632 ± 0.055 | 132.5 ± 3.1 | 1.919 ± 0.163 | 39.9 ± 6.3 |
| French | High yield | BSY 100 | 2.97 ± 0.11 | 0.378 ± 0.013 | 100.2 ± 0.2 | 1.926 ± 0.162 | 27.7 ± 3.4 |
|  |  | Baguette 9 | 3.50 ± 0.03 | 0.498 ± 0.019 | 96.7 ± 1.2 | 2.141 ± 0.015 | 19.4 ± 0.8 |
|  |  | Baguette 18 | 3.64 ± 0.06 | 0.524 ± 0.003 | 100.7 ± 5.3 | 2.257 ± 0.163 | 22.0 ± 0.9 |
|  |  | Baguette 19 | 3.48 ± 0.06 | 0.506 ± 0.003 | 88.5 ± 2.5 | 2.192 ± 0.185 | 13.6 ± 2.0 |
|  |  | Baguette 31 | 3.48 ± 0.14 | 0.475 ± 0.062 | 83.2 ± 2.2 | 2.095 ± 0.037 | 7.6 ± 1.1 |
|  |  | SNR Nogal | 3.63 ± 0.09 | 0.498 ± 0.021 | 78.7 ± 3.2 | 2.447 ± 0.019 | 8.1 ± 2.4 |
|  | Low Yield | BSY 200 | 3.55 ± 0.06 | 0.550 ± 0.005 | 93.2 ± 5.3 | 2.259 ± 0.209 | 16.9 ± 0.1 |
|  |  | Klein Atlas | 3.01 ± 0.26 | 0.449 ± 0.019 | 102.7 ± 0.7 | 1.564 ± 0.203 | 20.3 ± 5.2 |
|  |  | Klein Centauro | 3.28 ± 0.08 | 0.534 ± 0.015 | 99.0 ± 0.5 | 2.132 ± 0.107 | 24.6 ± 2.9 |
|  |  | Baguette 10 | 3.77 ± 0.04 | 0.560 ± 0.037 | 88.0 ± 1.0 | 2.271 ± 0.230 | 7.3 ± 6.0 |
|  |  | Baguette 21 | 3.66 ± 0.04 | 0.462 ± 0.016 | 80.7 ± 1.2 | 2.315 ± 0.197 | 7.1 ± 3.3 |
|  |  | Sinvalocho | 2.93 ± 0.02 | 0.493 ± 0.006 | 107.5 ± 1.0 | 1.526 ± 0.041 | 24.0 ± 1.8 |

Stem diameter, stem wall thickness, plant height, dry spike weight, and lodging susceptibility index for the 36 wheat genotypes assessed during the 2014 season. Data represents the mean and standard error of 3 replicates. ^a^ Single determination.
