# Supplementary material for: Towards an ideotype for food-fuel dual-purpose wheat in Argentina with focus on biogas production
Source: Biotechnol Biofuels. 2021 Apr 5;14:85. doi: 10.1186/s13068-021-01941-x (PMC8022367; doi:10.1186/s13068-021-01941-x)
Supplement: Supplementary file 2 — Additional file 2. Concentration of main components of wheat straw. [file 13068_2021_1941_MOESM2_ESM.docx]

**Additional file 2: Concentration of main components of wheat straw**

| Origin | Yield group | Genotype | Cellulose  (mg/mg DW) | Hemicellulose  (mg/mg DW) | Lignin  (mg/mg DW) |
| --- | --- | --- | --- | --- | --- |
| CIMMYT | High Yield | Buck AGP Fast | 0.39 | 0.30 | 0.17 |
|  |  | Don Mario Arex | 0.42 | 0.32 | 0.16 |
|  |  | Don Mario Atlax | 0.39 | 0.32 | 0.18 |
|  |  | INIA Centinela | 0.43 | 0.31 | 0.19 |
|  |  | Klein Don Enrique | 0.42 | 0.32 | 0.21 |
|  |  | Sursem LE 2331 | 0.39 | 0.31 | 0.15 |
|  | Low Yield | ACA 907 | 0.40 | 0.30 | 0.16 |
|  |  | BIOINTA 1003 | 0.37 | 0.27 | 0.13 |
|  |  | BIOINTA 3004 | 0.38 | 0.29 | 0.13 |
|  |  | Buck Puelche | 0.37 | 0.31 | 0.14 |
|  |  | Klein Cacique | 0.47 | 0.33 | 0.20 |
|  |  | Klein Yarara | 0.40 | 0.30 | 0.17 |
| *Criollos* | High Yield | BIOINTA 1000 | 0.41 | 0.30 | 0.13 |
|  |  | Buck 75 Aniversario | 0.42 | 0.29 | 0.14 |
|  |  | Buck Baqueano | 0.42 | 0.30 | 0.12 |
|  |  | Buck Guapo | 0.43 | 0.31 | 0.14 |
|  |  | Buck Ranquel | 0.39 | 0.32 | 0.14 |
|  |  | Don Mario Themix | 0.39 | 0.30 | 0.18 |
|  | Low Yield | Barletta 77 | 0.37 | 0.27 | 0.13 |
|  |  | Buck Naposta | 0.43 | 0.30 | 0.15 |
|  |  | INIA Condor | 0.37 | 0.29 | 0.17 |
|  |  | Klein Impacto | 0.41 | 0.30 | 0.19 |
|  |  | Klein Rendidor | 0.38 | 0.29 | 0.18 |
|  |  | Oleata Artillero | 0.43 | 0.32 | 0.17 |
| French | High Yield | BSY 100 | 0.41 | 0.33 | 0.14 |
|  |  | Baguette 9 | 0.44 | 0.32 | 0.14 |
|  |  | Baguette 18 | 0.43 | 0.30 | 0.16 |
|  |  | Baguette 19 | 0.40 | 0.30 | 0.15 |
|  |  | Baguette 31 | 0.39 | 0.31 | 0.14 |
|  |  | SNR Nogal | 0.36 | 0.28 | 0.15 |
|  | Low Yield | BSY 200 | 0.37 | 0.28 | 0.13 |
|  |  | Klein Atlas | 0.44 | 0.32 | 0.19 |
|  |  | Klein Centauro | 0.36 | 0.29 | 0.17 |
|  |  | Baguette 10 | 0.40 | 0.29 | 0.16 |
|  |  | Baguette 21 | 0.40 | 0.30 | 0.14 |
|  |  | Sinvalocho | 0.43 | 0.32 | 0.17 |

Cellulose, hemicellulose, and lignin determined in oven-dried samples of wheat stem of the 36 genotypes assessed during the 2017 season. Data represent a single determination.
