# Supplementary material for: Towards an ideotype for food-fuel dual-purpose wheat in Argentina with focus on biogas production
Source: Biotechnol Biofuels. 2021 Apr 5;14:85. doi: 10.1186/s13068-021-01941-x (PMC8022367; doi:10.1186/s13068-021-01941-x)
Supplement: Supplementary file 3 — Additional file 3. Kinetic parameters of biogas production and methane (CH4) percentage for wheat genotypes. [file 13068_2021_1941_MOESM3_ESM.docx]

**Additional file 3: Kinetic parameters of biogas production and methane (CH_4_) percentage for wheat genotypes**

| Origin | Yield group | Genotype | 2014 | |  |  | 2017 | |  |
| --- | --- | --- | --- | --- | --- | --- | --- | --- | --- |
|  |  |  | **B_max_**  (cm^3^/g VS) | ***k***  (day^-1^) | **CH_4_**  (%) | **Methane yield**  **(m^3^/ha)** | **B_max_**  (cm^3^/g VS) | ***k***  (day^-1^) | **CH_4_**  (%) |
| CIMMYT | High yield | Buck AGP Fast | 419.6 ± 2.1 | 0.102 ± 0.006 | 56.4 ± 1.5 | 1661 ± 14 | 392.9 ± 3.8 | 0.107 ± 0.001 | 55.8 ± 0.4 |
|  |  | Don Mario Arex | 455.2 ± 13.6 | 0.099 ± 0.001 | 54.6 ± 1.6 | 1893 ± 40 | 446.9 ± 0.2 | 0.132 ± 0.002 | 54.3 ± 0.4 |
|  |  | Don Mario Atlax | 398.4 ± 2.7 | 0.111 ± 0.001 | 56.1 ± 1.7 | 1717 ± 20 | 422.8 ± 5.1 | 0.139 ± 0.002 | 55.8 ± 0.4 |
|  |  | INIA Centinela | 419.0 ± 4.0 | 0.106 ± 0.003 | 56.1 ± 1.7 | 1794 ± 17 | 444.1 ± 14.1 | 0.124 ± 0.005 | 56.2 ± 0.0 |
|  |  | Klein Don Enrique | 435.4 ± 1.7 | 0.118 ± 0.002 | 55.5 ±1.1 | 1858 ± 18 | 435.1 ± 16.6 | 0.125 ± 0.003 | 55.8 ± 0.4 |
|  |  | Sursem LE 2331 | 419.3 ± 13.4 | 0.135 ± 0.009 | 56.0 ± 0.9 | 1879 ± 85 | 453.6 ± 4.0 | 0.141 ± 0.002 | 55.8 ± 0.4 |
|  | Low yield | ACA 907 | 391.8 ± 3.7 | 0.153 ± 0.005 | 55.5 ± 1.1 | 1067 ± 10 | 400.2 ± 15.9 | 0.121 ± 0.002 | 55.0 ± 0.4 |
|  |  | BIOINTA 1003 | 432.4 ± 2.5 | 0.134 ± 0.002 | 55.1± 2.1 | 1583 ± 18 | 411.2 ± 1.4 | 0.144 ± 0.007 | 56.2 ± 0.0 |
|  |  | BIOINTA 3004 | 414.2 ± 0.4 | 0.127 ± 0.001 | 54.6 ± 1.6 | 1030 ± 20 | 412.8 ± 8.1 | 0.129 ± 0.003 | 55.8 ± 1.1 |
|  |  | Buck Puelche | 445.7 ± 11.7 | 0.123 ± 0.003 | 54.6 ± 1.6 | 1419 ± 37 | 432.2 ± 10.6 | 0.128 ± 0.005 | 56.2 ± 1.5 |
|  |  | Klein Cacique | 413 ± 9.9 | 0.113 ± 0.002 | 56.5 ± 0.6 | 1330 ± 26 | 427 ± 5.0 | 0.109 ± 0.002 | 56.6 ± 0.3 |
|  |  | Klein Yarara | 411.9 ± 4.6 | 0.103 ± 0.009 | 55.5 ± 1.1 | 998 ± 15 | 424.4 ± 11.5 | 0.122 ± 0.005 | 56.2 ± 0.0 |
| *Criollos* | High yield | BIOINTA 1000 | 404.2 ± 9.0 | 0.135 ± 0.003 | 54.0 ±1.1 | 1855 ± 33 | 407.7 ± 6.6 | 0.112 ± 0.036 | 56.2 ± 0.0 |
|  |  | Buck 75 Aniversario | 399.5 ± 2.8 | 0.119 ± 0.002 | 55.5 ± 1.1 | 1901 ± 6 | 433.0 ± 6.4 | 0.145 ± 0.005 | 54.7 ± 0.0 |
|  |  | Buck Baqueano | 433.7 ± 3.2 | 0.130 ± 0.001 | 55.5 ± 1.1 | 2288 ± 37 | 432.8 ± 3.8 | 0.139 ± 0.005 | 55.8 ± 0.4 |
|  |  | Buck Guapo | 459.1 ± 0.8 | 0.127 ± 0.003 | 55.5 ± 1.1 | 3113 ± 19 | 464.7 ± 12.9 | 0.112 ± 0.001 | 55.0 ± 0.4 |
|  |  | Buck Ranquel | 404.3 ± 5.8 | 0.129 ± 0.004 | 55.1 ± 1.4 | 2041 ± 20 | 461.8 ± 4.1 | 0.125 ± 0.003 | 55.0 ± 0.4 |
|  |  | Don Mario Themix | 436.3 ± 9.7 | 0.119 ± 0.007 | 55.1± 1.4 | 1787 ± 22 | 454.8 ± 3.8 | 0.141 ± 0.003 | 55.0 ± 0.4 |
|  | Low yield | Barletta 77 | 388.2 ± 1.8 | 0.109 ± 0.005 | 55.6 ± 1.9 | 1834 ± 23 | 425.0 ± 6.8 | 0.139 ± 0.006 | 55.8 ± 0.4 |
|  |  | Buck Naposta | 378.3 ± 8.3 | 0.128 ± 0.004 | 56.1 ± 1.7 | 1445 ± 31 | 469.0 ± 21.5 | 0.079 ± 0.009 | 55.0 ± 0.4 |
|  |  | INIA Condor | 444.9 ± 14.8 | 0.099 ± 0.005 | 55.5 ± 1.1 | 1538 ± 51 | 415.4 ± 2.7 | 0.151 ± 0.002 | 56.6 ± 0.3 |
|  |  | Klein Impacto | 368.1 ± 10.2 | 0.114 ± 0.002 | 57.0 ± 1.2 | 1255 ± 23 | 473.5 ± 21.8 | 0.108 ± 0.005 | 55.8 ± 0.4 |
|  |  | Klein Rendidor | 410.3 ± 5.4 | 0.130 ± 0.007 | 56.5 ± 0.6 | 1397 ± 18 | 412.2 ± 0.3 | 0.146 ± 0.002 | 56.2 ±0.0 |
|  |  | Oleata Artillero | 443.8 ± 6.3 | 0.129 ± 0.007 | 55.5 ± 1.1 | 1545 ± 15 | 441.5 ± 4.9 | 0.110 ± 0.002 | 55.8 ± 1.1 |
| French | High Yield | BSY 100 | 438.2 ± 16.9 | 0.132 ± 0.005 | 54.6 ± 1.6 | 2062 ± 61 | 461.5 ± 24.7 | 0.122 ± 0.002 | 55.0 ± 0.4 |
|  |  | Baguette 9 | 400.4 ± 10.1 | 0.138 ± 0.002 | 55.1± 1.4 | 1694 ± 43 | 436.4 ± 5.4 | 0.133 ± 0.001 | 55.0 ± 0.4 |
|  |  | Baguette 18 | 436.0 ± 0.3 | 0.107 ± 0.002 | 55.5 ± 1.1 | 1871 ± 15 | 429.4 ± 13.9 | 0.109 ± 0.013 | 55.8 ± 0.4 |
|  |  | Baguette 19 | 421.6 ± 6.9 | 0.123 ± 0.001 | 55.1 ± 1.4 | 1678 ± 20 | 411.5 ± 6.8 | 0.123 ± 0.009 | 56.6 ± 0.3 |
|  |  | Baguette 31 | 436.9 ± 3.2 | 0.129 ± 0.001 | 56.1 ± 1.7 | 2111 ± 15 | 447 ± 17.2 | 0.108 ± 0.013 | 55.8 ± 0.4 |
|  |  | SNR Nogal | 426.8 ± 3.3 | 0.134 ± 0.001 | 56.5 ± 0.6 | 2247 ± 37 | 419.9 ± 8.6 | 0.163 ± 0.014 | 56.6 ± 0.3 |
|  | Low Yield | BSY 200 | 410.8 ± 2.4 | 0.133 ± 0.004 | 55.5 ± 1.1 | 1323 ± 8 | 401.6 ± 6.6 | 0.122 ± 0.006 | 56.6 ± 0.3 |
|  |  | Klein Atlas | 422.4 ± 9.6 | 0.118 ± 0.004 | 55.5 ± 1.1 | 1812 ± 55 | 453.2 ± 8.2 | 0.113 ± 0.002 | 56.2 ± 0.0 |
|  |  | Klein Centauro | 423.7 ± 12.7 | 0.116 ± 0.005 | 56.1 ± 1.7 | 1939 ± 50 | 421.6 ± 1.41 | 0.140 ± 0.002 | 55.0 ± 1.2 |
|  |  | Baguette 10 | 427.1 ± 2.7 | 0.134 ± 0.005 | 55.6 ± 1.9 | 1353 ± 14 | 420.2 ± 11.4 | 0.142 ± 0.003 | 56.6 ± 0.3 |
|  |  | Baguette 21 | 423.4 ± 0.4 | 0.144 ± 0.003 | 55.1 ± 2.1 | 1378 ± 12 | 429.9 ± 3.1 | 0.143 ± 0.004 | 55.8 ± 0.4 |
|  |  | Sinvalocho | 451.1 ± 19.1 | 0.097 ± 0.001 | 55.5 ± 1.1 | 1563 ± 66 | 466.3 ± 16.2 | 0.106 ± 0.002 | 55.0 ± 0.4 |

Best fitting values for B_max_ and *k*, and methane content of biogas for the 36 wheat genotypes assessed during the 2014 and 2017 seasons. Methane content was determined after cumulative biogas reached a plateau. Methane yield was determined during season 2014. Data represent the mean ± standard error of 2 replicates.
